# Electrospun Fibers Derived from Peptide Coupled Amphiphilic Copolymers for Dorsal Root Ganglion (DRG) Outgrowth

**DOI:** 10.3390/gels7040196

**Published:** 2021-11-04

**Authors:** Na Qiang, Wensheng Lin, Xingwu Zhou, Zhu Liu, Ming Lu, Si Qiu, Shuo Tang, Jixiang Zhu

**Affiliations:** 1 School of Chemistry and Materials Engineering, Huizhou University, Huizhou 516007, China; qiangna93@163.com (N.Q.); lz890927@126.com (Z.L.); reggi_lu@hzu.edu.cn (M.L.); qiusi250@gmail.com (S.Q.); 2 Department of Biomedical Engineering, School of Basic Medical Sciences, Guangzhou Medical University, Guangzhou 511436, China; heinrichlws@stu.gzhmu.edu.cn; 3 Department of Pharmaceutical Sciences, University of Michigan, Ann Arbor, MI 48109, USA; xwzhou@med.umich.edu; 4 Department of Orthopaedics, The Eighth Affiliated Hospital, Sun Yat-sen University, Shenzhen 517000, China; 5 The Sixth Affiliated Hospital of Guangzhou Medical University, Qingyuan 511518, China

**Keywords:** electrospun, functional monomer, isoleucine-lysine-valine-alanine-valine, dorsal root ganglion

## Abstract

Developing scaffolds with appropriate mechanical/structural features as well as tunable bioactivities are indispensable in the field of tissue engineering. This study focused on one such attempt to electrospin the copolymer of L-lactic acid (L-LA) and functional monomer (3(S)- [(benzyloxycarbony)methyl]-1,4-dioxane-2,5-dione, BMD) with small peptide modifications for the purpose of neural tissue engineering. Scanning Electron Microscopy (SEM) micrographs showed fabricated electrospun copolymer as porous and uniform nanofibrous materials with diameter in the range of 800–1000 nm. In addition, the modified scaffolds displayed a lower contact angle than poly(L-lactide) (PLLA) indicating higher hydrophilicity. To further incorporate the bioactive functions, the nanofibers were chemically coupled with small peptide (isoleucine-lysine-valine-alanine-valine, IKVAV). The incorporation of IKVAV onto the electrospun fiber was confirmed by X-ray photoelectron spectroscopy (XPS) and such incorporation did not affect the surface morphology or fiber diameters. To demonstrate the potential of applying the designed scaffolds for nerve regeneration, dorsal root ganglion (DRG) neurons were cultured on the nanofibers to examine the impact on neurite outgrowth of DRGs. The results indicated that the fabricated nanofibrous matrix with small peptide might be a potential candidate for neural tissue engineering.

## 1. Introduction

Electrospinning is used commonly to fabricate scaffolds for tissue engineering. It is capable of fabricating fibers in the range of nanoscale. Electrospun fibers are also being examined as extracellular matrix (ECM)-mimicking substrates in tissue engineering [1,2,3]. Many synthetic polymers can be electrospun into nanofibers, such as poly(ε-caprolactone) (PCL) [4,5], poly(lactic-co-glycolic acid) (PLGA) [6,7], and poly(L-lactic acid) (PLLA) [8,9]. The synthetic copolymer of L-lactide acid (L-LA) and ε-caprolactone(ε-CL), L-lactide acid (L-LA) and glycolic acid (GA) (i.e., PLLC, PLGA) are good scaffold candidates based on its biological compatibility [10,11]. In the meantime, they can be made into nanofibers by electrospinning [12,13] to better direct cell migration and tissue regeneration due to their unique structural and mechanical features [14]. Specifically, neural cells can respond to topographical cues, however, the electrospun fibers from synthetic polymers generally lack the bioactive molecules to efficiently promote cell growth and tissue formation [15,16]. Therefore, biomimetic modification of electrospun fibers have gained interest in tissue engineering and a variety of techniques have been developed for this purpose. Such as peptides on the surface of the electrospun nanofibers are capable of controlled release for antimicrobial abilities [17]. Ion irradiation [18], plasma treatment [19,20], surface coating [21,22] and material hydrolysis [23] have been used to functionalize polymer surface. Especially, the surface can be modified by small peptide as biologically cues for cell migration and differentiation [24,25]. Laminin-derived peptides (RGD, IKVAV, YIGSR et al.) have been shown to promote cell adhesion and induce neurite outgrowth of neural cells [26,27].

Recent reports have tried to postprocess nanofiberous substrates from electrospinning for attachment of biologically active peptides [28]. For example, the Becker groups utilized DIBI-end functionalized poly(ε-caprolactone) and modified the nanofibers with peptides after electrospinning. However, the process needs solvent to dissolve polymers and the biological applications was limited because of the residual solvent used [29].

Nanofibers introduce new reactive functionality, particularly surface-immobilized functional groups. This allowed for the coupling reaction to covalently immobilize biologically active peptides (i.e., RGD) onto the fiber surface. Following RGD modification, it was found that the peptide remained active to induce adhesion and spreading of the cells [30]. It is well-known that cells can respond to biologically active molecules, such as RGD, YIGSR and IKVAV. In particular, neuronal cells are sensitive to laminin-derived peptides and can undergo axonal elongation [31].

Here, we synthesized and characterized synthetic polymeric nanofibers comprised of functional polyester with carboxyl groups via electrospinning. Electrospinning was used to create nanofiber matrices with fiber diameters near the size of the nanotopography, which has been shown to promote neural cell differentiation and neurite extension. The functional polyester is presented with carboxyl groups to allow for chemical coupling with bioactive molecules, including a small peptide (i.e., IKVAV) on to the electrospun fibers. Following small peptide modification, we investigate DRGs growth in response to the surface bioactive molecules modified nanofibers.

## 2. Results and Discussion

### 2.1. Morphology of Electrospun PLLA and PLB-g-IKVAV Fibers

**Figure 1:**
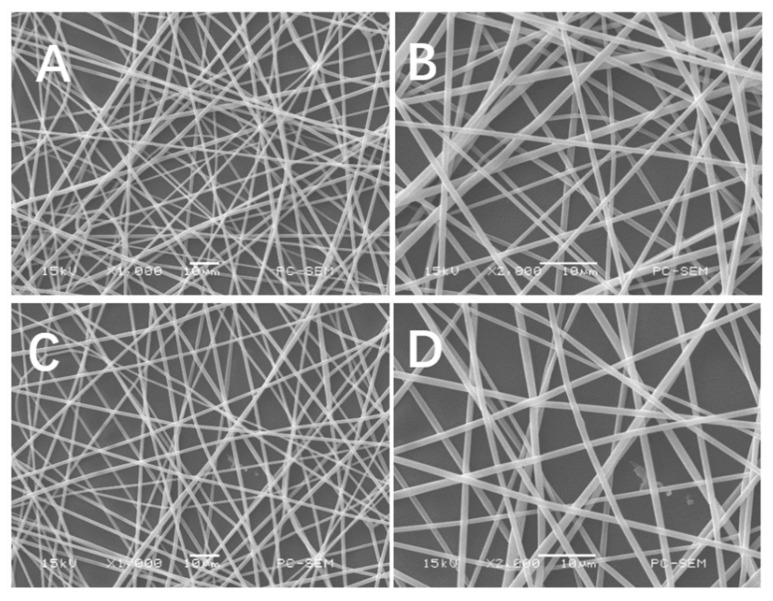
The SEM morphology of electrospun PLLA (**A**,**B**) and PLB-g-IKVAV (**C**,**D**) from total 15% solution. Magnifications of PLLA (**A**,**B**) and PLB-g-IKVAV (**C**,**D**) were displayed. Uniform polymeric fibers with and without peptide modification were gained at nanoscale from electrospinning.

**Figure 2:**
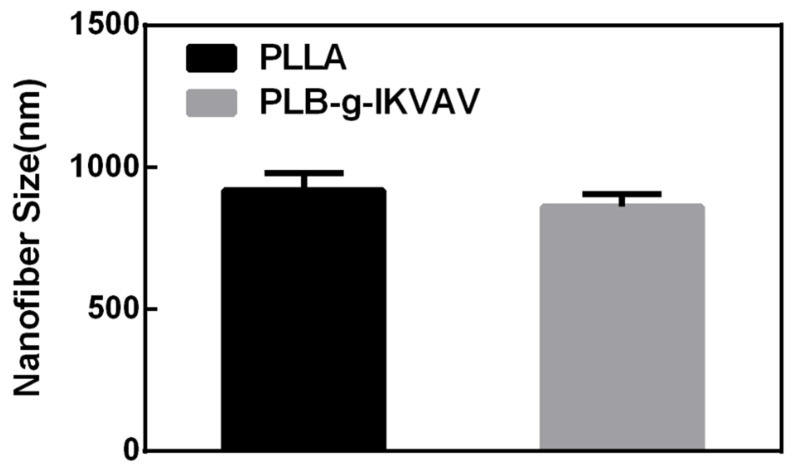
Mean diameter (±SD) variations of electrospun PLLA and PLB-g-IKVAV fibers were interpretated from randomly selected 20 nanofibers in the SEM images. Nanofibers with and without peptide modifications displayed no significant changes in size.

First, the functional polymer was synthesized by the ring-opening polymerization of L-LA and BMD catalyzed by stannous octoate using dodecanol as the initiator. Poly(L-LA-co-BMD) (PLB) and PLLA were further electrospun into micro/nano fibers for further characterizations. In the electrospinning system, there are a number of parameters affecting fiber morphology, such as polymer concentration, applied voltage and the delivery rate of the polymer solution. Additionally, the solvent used to dissolve polymer has a significant effect on the spinnability of a polymer solution and the fiber morphology. Here, trifluoroethanol (TFEA) was used as a solvent to dissolve both PLB and PLLA with the concentration of the polymers as 15%. The electrospinning condition was optimized to have pumping rate of 1 mL/h under 15kV (for PLB). Faster pumping rate and/or higher voltage resulted in unsuccessful fiber formation. Morphological images of the electrospun PLB fibers was examined by SEM and are shown in Figure 1. In the SEM images, the lower pumping rate (0.5 mL/h) and voltage (12kV) produced fibers with smaller diameters. Fiber diameters analyzed from SEM images were ranged from 800 to 1000 nm (Figure 2). The addition of small peptide resulted in the decrease of fiber diameters, which is consistent with the results reported in other literature [32]. Therefore, the fibrous scaffolds emulating the size scale of the native ECM were successfully fabricated via electrospinning.

**Figure 3:**
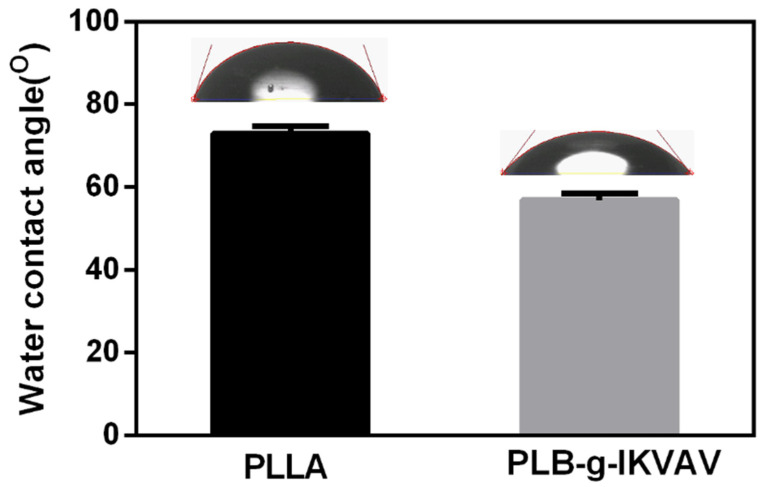
Contact angle of PLLA and PLB-g-IKVAV fibers (n = 4) were measured. Nanofibers with peptide modification had a smaller contact angle indicating higher hydrophilicity.

Water contact angle provides an indication of the hydrophilicity on the surface of the electrospinning substrates. Cell adhesion also depends on the wettability of the surface, since synthetic biocompatible polymers, such as PCL, PLLA and PLGA, are hydrophobic [2,6], which usually limits cell interactions with the matrix. Incorporating peptides such as IKVAV or YIGSR will likely improve the hydrophilicity of the surface and thus enhance the interactions with cell surface receptors. Given the fact that the cell membrane is negatively charged and some peptide possesses positively charged [33], further enhancing the cell adhesion properties of the polymeric substrates [34]. Water contact angles were measured on PLB-g-IKVAV and PLLA fibers and it was shown (Figure 3) that PLB-g-IKVAV fibers become more hydrophilic since the contact angle decreased. Therefore, it is likely that receptor specific interactions could improve cell surface interaction and adhesion.

**Figure 4:**
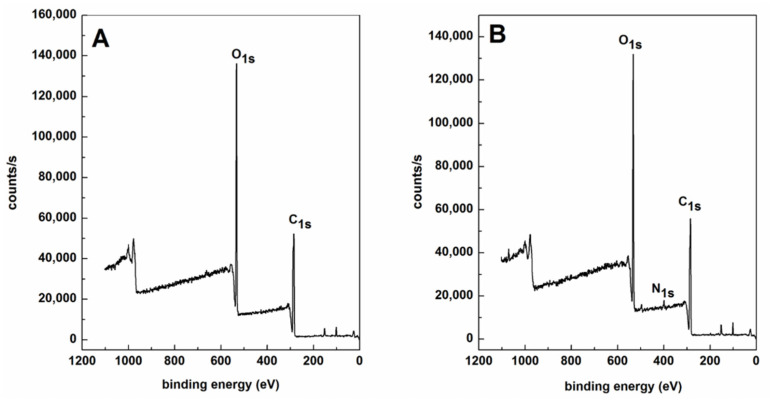
XPS signals of PLLA (**A**) and after coupling with IKVAV (**B**). The nitrogen signal come from the peptide modification indicating successful chemical conjugation.

Finally, PLB-g-IKVAV fibers were characterized by X-ray photoelectron spectroscopy (XPS) to determine nitrogen content and the results were shown in Figure 4 and Table 1. Nitrogen is a unique atom existing only after the fibers are peptide-modified. XPS wide scan of PLB-g-IKVAV and PLLA fibers was performed and the intensity of N1s was quantified. An increasing intensity of N1s (relative %) was observed, indicating the successful immobilization of IKVAV onto the PLB fibers. The percentage of nitrogen was corresponding to the amount of coupled IKVAV. PLLA fibers serve as the negative control since they do not contain nitrogen. The result confirmed the existence of IKVAV on the PLB membrane.

### 2.2. DRGs Proliferation Studies on Electrospun PLLA and PLB-g-IKVAV Fiber Sheets

**Figure 5:**
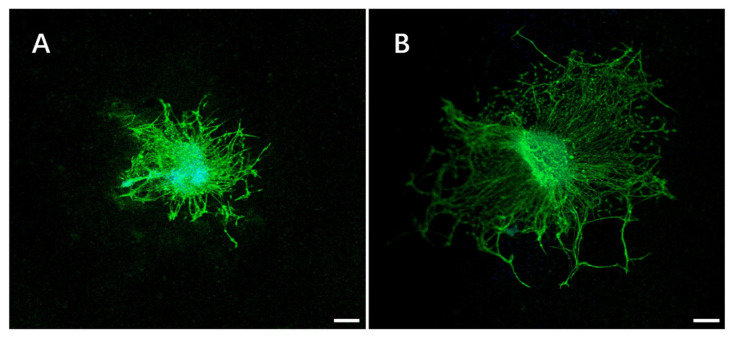
Fluorescence images of DRGs seeding on electrospun PLLA films (**A**) and PLB-g-IKVAV films (**B**) for 7d. Scale bar = 200 μm.

**Figure 6:**
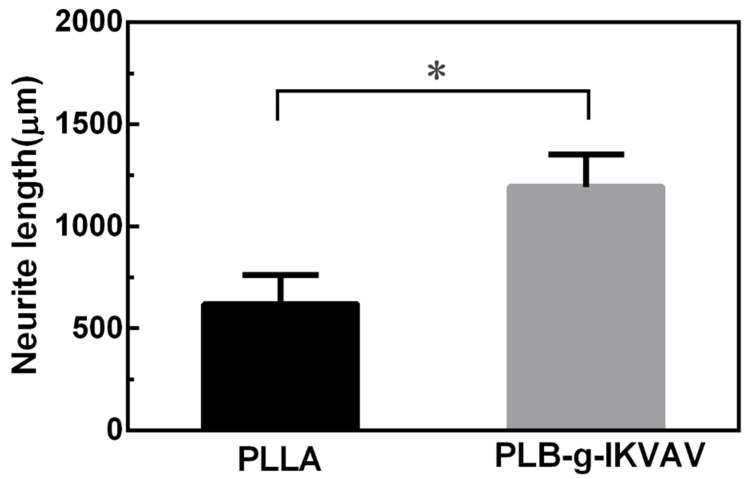
Quantification of the neurite length of DRGs seeded on different films for 7d interpretated from the fluorescence images (* *p* < 0.05).

To prove the bioactivity of the modified fibers in vitro, DRGs were implanted on the electrospinning films with IKVAV before the immunochemical staining. As shown in Figure 5, the functionalization matrix can affect neural progenitor differentiation and neurite extension. DRGs are especially sensitive to the IKVAV modifications and can undergo axonal elongation. The cells had distinctly different morphologies on the electrospinning films modified by small peptide. For the IKVAV group, the spreading area of DRGs was larger than the control. Average neurite length of DRGs cultured on functionalized films with IKVAV was significantly longer than that of cells cultured on the control samples (Figure 6). The result was similar to those of previous studies where it was found that the incorporation of small peptide improved neurite extension compared to control [31].

## 3. Conclusions

In conclusion, we report covalently immobilized IKVAV peptides on electrospun fibers which induce DRGs outgrowth. Functional monomer BMD was selected due to the presence of carboxyl groups allowing for further functionalization, and it was copolymerized with L-LA. The copolymer was made into nanofibers by electrospinning in an effort to mimic ECM. After attaching IKVAV, we investigate DRGs growth in response to the small peptide. In our system, electrospun fibers with small peptide incorporation promote neural outgrowth. All the results demonstrate that coupling with IKVAV into PLB improves nerve regeneration, thus we believe that PLB-g-IKVAV have potential applications in nerve regeneration. Furthermore, the technology presented is modularly designed and can allow for selecting appropriate polymers and distinct peptide signals. Combining both together, it is possible to design bioactive scaffolds with different applications or for different tissue regeneration.

## 4. Materials and Methods

### 4.1. Materials

Poly(L-LA-co-BMD) (PLB) copolymers were synthesized as previous work [35]. All solvents used in this study were purchased from Guangzhou Chemical Reagent Factory. These chemicals were used as received without further purification.

### 4.2. Electrospinning

Functional monomer (BMD) was synthesized by L-aspartic acid through a four-step reaction. Then the copolymer was synthesized by ring-opening bulk polymerization of L-LA and BMD using dodecanol as initiator and Sn(Oct)_2_ as catalyst. The method was described in previous work [35]. The feed ratio, the Mn and the distribution of the copolymer was 95:5 (L-LA: BMD), 1.1 × 10^5^ and 1.12.

PLB solution at a concentration of 15% *w/v* was prepared at room temperature by dissolving the polymer in TFEA. The electrospinning was performed as follow: the electrospinning was equipped with a high voltage statitron (12kV). The solution flow rate was 0.5 mL/h and collecting distance was 10 cm at 25–28 °C. To prepare fibrous PLB films for cell culture, cover glasses with a diameter of 15 mm were placed on the plate to deposit. The samples were dried in a vacuum oven for over 2 days to remove solvent residue for further application.

### 4.3. IKVAV Conjugation onto Electrospun Polymer Fibers

Conjugation of peptide onto polymer was performed by using N, N’-dicyclohexylcarbodiimide/N-hydroxysuccinimide/ (DCC/NHS) chemistry [35]. The electrospun polymer fibers were then immersed in 1.0 mg/mL small peptide (IKVAV) solution after drying. The electrospinning films were soaked for 12 h with mild agitation and washed three times with phosphate-buffered saline (PBS) to remove unreacted small peptides.

### 4.4. Electrospun Fiber Characterization

The electrospun fibers of polymer were sputter coated with gold and visualized by Scanning Electron Microscopy (SEM) (SEM, JSM-6380LA Analytical SEM, JEOL Ltd., Tokyo, Japan) operated at an accelerating voltage of 15 kV. Fiber diameters were measured using Image J software (National Institutes of Health, Bethesda, MD, USA). At least 100 filaments of each sample from different SEM images were analyzed.

### 4.5. Surface Characterization

The surface atomic analysis of neat PLLA fibers and PLB-g-IKVAV were investigated by contact angles (CA) and X-ray photoelectron spectroscopy (XPS). The contact angles of water over the surface of films were measured with a VCA-Optima Surface Analysis System (AST Product, Inc., Billerica, MA, USA). At least 15 independent measurements were performed per each treatment. XPS was recorded on an AXISHSi spectrometer (Kratos Analytical Ltd., Stretford, UK) employing the excitation of a non-monochromatized Ka X-ray source (1486.7 eV). The core level spectra were obtained at a photoelectron take-off angle of 90° (measured with respect to the sample surface).

### 4.6. DRG Neurite Length

DRGs were placed on the matrix and incubated in medium containing B27 neural supplement (Invitrogen, Carlsbad, CA, USA), Neurobasal media (Invitrogen, Carlsbad, CA, USA), 1% penicillin streptomycin (Sigma Aldrich, St Louis, MO, USA) and 2 mM L-glutamine (Sigma Aldrich, St Louis, MO, USA). The DRGs were fixed in 4% paraformaldehyde at 4 °C after 7 d culturing, and permeabilized with 0.3% Triton X-100 for 5 min. Non-specific binding was blocked with 10% goat serum and 1% bovine serum albumin (BSA) for 1 h at room temperature. The DRGs were incubated with rabbit anti-neurofilament 200 (NF200) antibody. Alexa Fluor 488-conjugated goat anti-rabbit IgG was added after washing with PBS.

The samples were examined using a Zeiss LSM 710 laser scanning confocal microscope (LSCM, Zeiss, Oberkochen, Germany). Neurite outgrowth analysis was determined with image analysis software (ImageJ, National Institutes of Health, Bethesda, MD, USA). Results were expressed as mean ± standard deviation. One-way analysis of variance was used to test for statistical significance, and *p* < 0.05 was considered as statistically significant.

## Figures and Tables

**Table 1:** Atomic ratios carbon, oxygen, and nitrogen on the surface of PLLA and couple with small peptide (IKVAV) as determined by X-ray photoelectron spectrometry.

Samples	C Atomic Concentration(%)	N Atomic Concentration(%)	O Atomic Concentration(%)
PLLA	57.22	0	40.73
PCB-IKVAV	57.11	1.83	37.9

## Data Availability

Not applicable.
